# Signalling Through Retinoic Acid Receptors is Required for Reprogramming of Both Mouse Embryonic Fibroblast Cells and Epiblast Stem Cells to Induced Pluripotent Stem Cells

**DOI:** 10.1002/stem.1926

**Published:** 2015-04-23

**Authors:** Jian Yang, Wei Wang, Jolene Ooi, Lia S. Campos, Liming Lu, Pentao Liu

**Affiliations:** ^1^ Wellcome Trust Sanger InstituteHinxtonCambridgeUnited Kingdom; ^2^ Shanghai Institute of ImmunologyShanghai Jiaotong University School of Medicine280 South Chongqing RoadShanghai200025China

**Keywords:** Reprogramming, Induced pluripotent stem cells, Retinoic acid receptors, Retinoic acid receptor gamma, Liver receptor homolog‐1, β‐Catenin, Epiblast stem cells

## Abstract

We previously demonstrated that coexpressing retinoic acid (RA) receptor gamma and liver receptor homolog‐1 (LRH1 or NR5A2) with OCT4, MYC, KLF4, and SOX2 (4F) rapidly reprograms mouse embryonic fibroblast cells (MEFs) into induced pluripotent stem cells (iPSCs). Here, we further explore the role of RA in reprogramming and report that the six factors (6F) efficiently and directly reprogram MEFs into integration‐free iPSCs in defined medium (N2B27) in the absence of feeder cells. Through genetic and chemical approaches, we find that RA signalling is essential, in a highly dose‐sensitive manner, for MEF reprogramming. The removal of exogenous RA from N2B27, the inhibition of endogenous RA synthesis or the expression of a dominant‐negative form of RARA severely impedes reprogramming. By contrast, supplementing N2B27 with various retinoids substantially boosts reprogramming. In addition, when coexpressed with LRH1, RA receptors (RARs) can promote reprogramming in the absence of both exogenous and endogenously synthesized RA. Remarkably, the reprogramming of epiblast stem cells into embryonic stem cell‐like cells also requires low levels of RA, which can modulate Wnt signalling through physical interactions of RARs with β‐catenin. These results highlight the important functions of RA signalling in reprogramming somatic cells and primed stem cells to naïve pluripotency. Stem Cells *2015;33:1390–1404*

## Introduction

Reprogramming somatic cells to induced pluripotent stem cells (iPSCs) with defined genetic factors, namely OCT4, MYC, KLF4, and SOX2 (4F) 1, not only has changed our understanding of cell differentiation and dedifferentiation but also holds great promise for regenerative medicine. This approach has been proven to be successful in various mammalian species, such as mouse, rat, and human 2, 3, 4. Nonetheless, somatic reprogramming remains an inefficient process. The use of integrative genetic factors in standard 4F reprogramming also carries the risk of ectopic reactivation. Efficient nonintegrative reprogramming strategies or the use of small molecules are thus desired. Supplementation with valproic acid and vitamin C can reduce aberrant epigenetic changes in iPSCs, and several small molecules can replace genetic factors, such as KLF4 and SOX2, in reprogramming 5, 6, 7. There have also been reports of successful small molecule‐mediated reprogramming in the complete absence of exogenous genetic factors 8, 9, 10. The molecular mechanisms underlying most of the small molecules used are not well defined. They probably act through signalling modulation and direct interaction with epigenetic modifiers. Indeed, the modulation of specific signalling pathways, such as Jak/Stat and Wnt, by small molecules has been shown to facilitate reprogramming 11, 12.

We found that retinoic acid (RA) signalling and RA receptors (RARs) facilitate reprogramming 13. Vitamin A (VA) is a natural small molecule that can exist in several forms, such as retinol, and has pleiotropic functions in development and disease 14. Retinol binds to retinol binding protein to form a complex in the blood, which interacts with the specific cell surface receptor STRA6 for cellular uptake. Retinol is further metabolized to all‐*trans* retinoic acid (ATRA) or 9‐*cis*‐retinoic acid (9cRA). The RA ligands bind two classes of nuclear receptors, RARs and retinoid X receptors (RXRs). Each class has three isoforms, α, β, and γ. ATRA can bind and activate both RARs and RXRs, while 9cRA is thought to predominantly interact with RXRs 15, 16, 17. The ligand‐bound receptors are then translocated to the nucleus and regulate target genes by recruiting coactivators, such as p160, to the retinoic acid response element (RARE) 18.

Although high concentrations of RA in embryonic stem cells (ESCs) induce the expression of repressors of key pluripotency genes, including *Oct4* 19, 20, and of lineage specific genes 18, 21, 22, we found that expression of a dominant‐negative form of RARA (RARA‐DN) impeded mouse embryonic fibroblast cell (MEF) reprogramming. In contrast, coexpression of 4F with retinoic acid receptor gamma (RARG) and liver receptor homolog‐1 (LRH1) (6F) remarkably increased reprogramming efficiency and dramatically shortened the reprogramming course 13. In this study, we aimed to (a) further explore RA signalling in reprogramming by focusing on the differential and highly dose‐dependent requirements of the ligands and the receptors in reprogramming MEFs and epiblast stem cells (EpiSCs) and (b) use the highly efficient 6F reprogramming to directly produce integration‐free naïve iPSCs in a defined medium.

## Materials and Methods

### Mouse Work

The housing and breeding of mice and experimental procedures using mice were designed according to the UK 1986 Animals Scientific Procedure Act and local institute ethics committee regulations.

### Reagents

Oct4, c‐Myc, Klf4, Sox2, Rarg, Rara, and Lrh1 cDNAs and the RARA‐DN were cloned into *piggyBac* (*PB*) transposons under the control of the CMV early enhancer/chicken β actin (CAG) promoter or the doxycycline (Dox)‐inducible TRE promoter (TRE).

For episomal expression, cDNAs of 4F and 2F were cloned into episomal vectors under the control of CAG promoter to construct two expression vectors: pCEP‐4F and pCEP‐2F.

We also used the *PB* transposase plasmid, pGL3‐RARE‐Luciferase (Addgene, Cambridge, MA, https://www.addgene.org, plasmid, 13458), pRL‐TK (Renilla luciferase control plasmid) (Promega, Madison, WI, http://www.promega.com), and TOPflash (T‐cell factor [TCF] reporter plasmid) (Merk Millipore, Darmstadt, Germany, http://www.emdmillipore.com, gift from Dr. Jason Wray and Prof. Austin Smith, University of Cambridge, U.K.).

The diagrams of these constructs and plasmids are listed in Supporting Information Figure S1. ATRA, 9cRA, retinol, citral, and IWR‐1 were purchased from Sigma (Gillingham, UK, https://www.sigmaaldrich.com/united‐kingdom.html), and CD437 and CD2665 were obtained from Tocris Biosciences (Abingdon, UK, http://www.tocris.com). PD0325901 (PD) and CHIR99021(CH) were obtained from Axon Medchem, (Groningen, The Netherlands, http://www.axonmedchem.com).

### Cell Culture

Mouse iPSCs were cultured in N2B27/2i/leukemia inhibitory factor (LIF) or 2i/LIF 23, 24 with slight modifications; Dulbecco's modified Eagle medium (DMEM)/F12, l‐glutamine, N2, B27 (Invitrogen, Paisley, UK, http://www.lifetechnologies.com/uk/en/home.html), 2‐mecaptoethanol, PD (1.0 μM), CH (3.0 μΜ), and LIF were included. The MEFs were derived from E13.5 mouse embryos (with a mixed 129S5/C57B6J background) and cultured in M10. Knockout DMEM (Invitrogen), 10% fetal bovine serum (Hyclone, Logan, Utah, https://promo.gelifesciences.com/gl/hyclone/index.html), l‐glutamine, penicillin/streptomycin, and 2‐mecaptoethanol were included in this medium. Mouse *Oct4‐GFP* EpiSCs (gift from Dr. Jenifer Nichols and Prof. Austin Smith, University of Cambridge, U.K.) were cultured on fibronectin‐coated plates in N2B27, activin (20.0 ng/ml) (R&D Systems, Minneapolis, MN, http://www.rndsystems.com/index.aspx), and fibroblast growth factor 2 (FGF2; 12.0 ng/ml) (Peprotech, Rocky Hill, NJ, http://www.peprotech.com/en‐US), as previously described 25.

### Reprogramming

To reprogram MEFs, vectors (in most experiments, 2.0 µg *PB* transposon, 2.0 µg 4F, or 1.0 µg 4F plus 1.0 µg 2F (6F), and 2.0 µg *PB* transposase plasmid) were first mixed with 1 × 10^6^ cells in OptiMEM (Invitrogen), and the cells were electroporated with Amaxa Nucleofector (Lonza, Basal, Switzerland, http://www.lonza.com). After electroporation, the cells were plated onto gelatinized 10 cm dishes in M10 for recovery for 24 hours. The cells were then washed with phosphate buffered saline (PBS) and switched to N2B27/LIF with or without VA or additional chemicals (or Dox if inducible reprogramming factors were used). The medium was changed every other day, and the emerging iPSC colonies were monitored under a microscope. At day 14, iPSC colonies were picked and expanded in 2i/LIF for further characterization.

For episomal vector reprogramming, the vectors were transfected into *Rex1‐GFP* MEFs, and the cells were allowed to recover for 24 hours before the medium was switched to N2B27/LIF. The cells were kept in N2B27/LIF for 12 days before the medium was changed to 2i/LIF for another 6 days. The colonies were then stained for alkaline phosphatase (AP) activity.

For EpiSC reprogramming, *Oct4‐GFP* EpiSCs cultured in a six‐well plate (approximately 90% confluent) were transfected with Lipofectamine 2000 (Invitrogen) using 1.0 µg transposon DNA (expressing either *Lrh1* or *Klf4*) and 2.0 µg transposase plasmid. Twenty‐four hours after transfection, the cells were plated at 1.5 × 10^5^ per well in N2B27/activin/FGF2 (AF) for another 24 hours. The medium was switched to 2i/LIF for approximately 8 days. On day 8, Oct4‐GFP^+^ iPSC colonies were scored under a fluorescence microscope.

### Polymerase Chain Reaction and Quantitative Real‐Time PCR

DNA was prepared with a DNA/RNA AllPrep Kit (Qiagen, Duesseldorf, Germany, http://www.qiagen.com) or a DNAreleasy kit (Anachem, Luton, UK, https://www.anachem.co.uk) from episomal iPSCs. Between 100.0 ng and 1.0 µg, DNA was used for polymerase chain reaction (PCR) amplification, using Readymix Extensor PCR master mix (Thermo Scientific, Hemel Hempstead, UK, http://www.thermoscientific.com/en/home.html). The primers are listed in Table S1 in the Supporting Information.

RNA was prepared with an RNeasy Kit (Qiagen). One microgram of RNA was used for reverse transcription (Qiagen), and the same primers were used to check for exogenous expression of *Oct4*, *c‐Myc*, *Klf4*, *Sox2*, *Rarg*, and *Lrh1*. Quantitative real‐time PCR (qRT‐PCR) was performed using Taqman Gene Expression Assays (Applied Biosystems). Gene expression was determined relative to *Gapdh* using the ΔCt method. All the qRT‐PCR reactions were performed in a 7900 Real‐time PCR system (Applied Biosystems, U.K.). The Taqman probes are listed in Supporting Information Table S2.

### Luciferase Assay

MEFs (1 × 10^6^) were cotransfected with pGL3RARE and pRL‐TK by electroporation. After transfection, the cells were plated into a gelatinized six‐well plate in M10 for 24 hours. The cells were split 1:9 into a 24‐well plate in N2B27/LIF with or without VA or other retinoids for 24 hours. The cells were collected, and luciferase activity was analyzed with Microluma plus (Berthold Technologie, Bad Wildbad, Germany, https://www.berthold.com/en). EpiSCs (1 × 10^6^) were cotransfected with pGL3‐RARE‐Luciferase and pRL‐TK with Lipofectamine 2000 in AF for 24 hours. The cells were split 1:9 into a 24‐well plate for another 24 hours in AF with or without VA or other retinoids for the luciferase assay.

For the TOPflash assay, 1 × 10^6^ EpiSCs were cotransfected with TOPflash and pRL‐TK with Lipofectamine 2000 in AF for 24 hours. The cells were split 1:9 into a 24‐well plate for another 24 hours in N2B27 without VA. Retinoids or CH was subsequently added for the luciferase assay.

### Cell Proliferation and Apoptosis Assay

MEFs (1 × 10^5^) were cultured in N2B27/LIF with or without VA or ATRA. The cells were counted every day for 6 days. The cell growth curve was plotted.

A total of 1.5 × 10^5^ EpiSCs were plated into six wells in AF without VA and incubated for 24 hours, and the medium was switched to 2iLIF with or without VA or ATRA at 10.0 or 0.1 nM. Apoptosis was monitored for 2 days with the PE Annexin V apoptosis detection kit (BD Pharmingen, San Diego, CA, https://www.bdbiosciences.com). Briefly, the cells were dissociated with Accutase, washed with PBS, resuspended in binding buffer at a concentration of 1 × 10^6^ cell per milliliter, stained with PE Annexin V and 7‐AAD, and analyzed by flow cytometry (BD Biosciences, San Jose, CA, http://www.bdbiosciences.com).

### Immunofluorescence

iPSCs were plated at 2 × 10^3^ onto four well plates in 2i/LIF. After 24 hours, the cells were washed with PBS, fixed in 4% paraformaldehyde (PFA), blocked and permeabilized with 1% bovine serum albumin and 3% serum in PBS with 0.3% Triton X‐100. The specimens were incubated with anti‐SSEA‐1 (BD Biosciences) or anti‐Nanog (Abcam, Cambridge, UK, http://www.abcam.com) antibodies at 4°C overnight. They were then rinsed and incubated with Alex594‐conjugated goat anti‐Mouse IgM and Alex594‐conjugated goat anti‐Rabbit IgG (Invitrogen) antibodies and counterstained with DAPI.

### In Vitro Differentiation

iPSCs were plated at 5 × 10^5^ in M10 in Petri dishes for 4 days. The cells were then dissociated with 0.05% Trypsin/EDTA and plated at 1 × 10^5^ in M10 in gelatinized six‐well plates for another 4 days. Then, the cells were fixed in 4% PFA to check for mesoderm and endoderm maker expression by immunofluorescence using antibodies to smooth muscle antigen (R&D Systems) and alpha fetal protein (R&D Systems). For neuronal differentiation, the cells were plated at 1–1.5 × 10^5^ in N2B27 in gelatinized six‐well plates, and the medium was changed every other day. At day 8, the cells were fixed and stained with an anti‐beta tubulin III (Tuj1) antibody (R&D Systems).

### Coimmunoprecipitation and Western Blot Analyses

Cells (5 × 10^6^) were plated in AF (no VA) for 24 hours, and the medium was switched to N2B27 (no VA) with 3.0 µM CH and 5.0 nM CD437 for 24 hours. Nuclear proteins were extracted with a NE‐PER nuclear and cytoplasmic extraction kit (Thermo Scientific), and protein levels were quantified with a BCA protein assay kit (Thermo Scientific). For Coimmunoprecipitation, 200.0 µg of nuclear protein was incubated with Protein G magnetic beads (Bio‐Rad, Hemel Hempstead, UK, http://www.bio‐rad.com) at 4°C for 1 hour, and the supernatant was incubated with anti‐RARG (Abcam) at 4°C overnight and then incubated with Protein G magnetic beads at 4°C for 1 hour. The beads were then washed and resuspended in SDS sample loading buffer. Western blots were performed according to a standard protocol. The primary antibodies were against β‐catenin (Sigma) and H3 (Cell Signaling, Denvers, MA, http://www.cellsignal.com).

### Teratoma and Chimeric Mice

For the teratoma assay, 1.0 × 10^6^ cells in 1.0 ml PBS were injected into two flanks of NSG mice. After 1 month, the tumors were collected and fixed in 37% formaldehyde for histology analysis.

Chimeras were produced by a standard microinjection protocol. Chimerism was estimated based on coat color given that the iPSCs were derived from MEFs of a mixed 129S5/C57B6J genetic background (dark furs), whereas the host blastocysts were from albino C57B6.

### Statistics

The data are presented as the mean ± standard deviation. Significance was determined using Student's unpaired *t* test with two‐tailed distribution. *p*‐Values less than .05 were considered significant.

## Results

### 6F Enables Efficient Direct Reprogramming of MEFs to iPSCs in N2B27/LIF

Reprogramming is primarily performed in medium containing fetal calf serum or on feeder cells, which may complicate the downstream investigation of the role of RA signalling in reprogramming. We thus set out to investigate the direct reprogramming of MEFs to iPSCs by 6F in a defined medium, specifically N2B27, which consists of DMEM/F12 with the two supplements, N2 and B27. N2 is primarily used to culture postmitotic neurons and contains insulin and apo‐transferrin 26, whereas B27 contains vitamins, hormones, proteins, and antioxidants 27. N2B27 medium supports mouse ESC self‐renewal and pluripotency with LIF and bone morphogenetic protein 4 28. As activation of STAT3 facilitates the reprogramming of MEFs and EpiSCs to ground state pluripotency 11, we also added LIF to N2B27.

**Figure 1:**
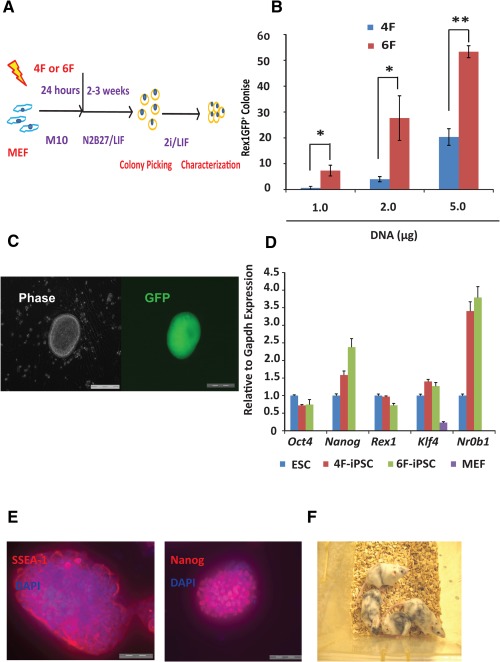
Reprogramming MEFs to iPSCs in N2B27 by 4F and 6F. **(A):** Diagram of the reprogramming strategy. After transfection, MEFs were recovered in M10 before being subsequently cultured in N2B27/LIF. Colonies were picked and cultured in N2B27/2i/LIF for characterization. **(B):** Effects of increased doses of reprogramming factors. GFP^+^ colonies from *Rex1‐GFP* MEFs were scored on day 18. *, *p* < .05; **, *p* < .01. **(C):** The morphology of a Rex1‐GFP^+^ 6F‐iPSC colony in 2i/LIF on day 10. Scale bar = 200 µm. **(D):** Quantitative real‐time PCR analysis of pluripotent gene expression in 4F‐ and 6‐iPSCs. The expression levels are shown relative to *Gapdh* and normalized to ESCs. **(E):** Immunostaining of 6F‐iPSCs for SSEA‐1 and Nanog. Scale bar = 100 µm. **(F):** Chimeric mice with a contribution (dark fur) from 6F‐iPSCs. The experiments were repeated at least three times, and error bars show standard deviations from the mean of triplicate measurements in one representative experiment. Abbreviations: DAPI, 4′,6‐diamidino‐2‐phenylindole; ESC, embryonic stem cell; GFP, green fluorescent protein; iPSC, induced pluripotent stem cell; LIF, leukemia inhibitory factor; MEF, mouse embryonic fibroblast cell.

We first transfected *Rex1‐GFP* reporter MEFs 13, 29 (1 × 10^6^) with *piggyBac* (*PB*) transposon vectors carrying either Dox‐inducible 4F (TRE‐4F) or 6F (TRE‐4F plus TRE‐RL, *Rarg* and *Lrh1*) (Fig. 1A and Supporting Information Fig. S1). As *Rex1* is expressed in ground state ESCs but not in primed EpiSCs 30, 31, the reactivation of green fluorescent protein (GFP) expression from *Rex1‐GFP* MEFs provides a convenient way to monitor the reprogramming kinetics and distinguish naïve iPSCs from partially reprogrammed cells. The transfected MEFs were plated on a gelatinized dish without feeder cells and allowed to recover in serum‐containing M10 medium before the medium was switched to N2B27/LIF plus Dox (Fig. 1A). In 6F reprogramming, the MEFs began to exhibit epithelial‐like morphology on day 5. Colonies emerged on day 6, and approximately 30% of them showed heterogeneous GFP signals on day 8, indicating successful *Rex1* reactivation (Supporting Information Fig. S2A). By contrast, in the 4F control, the earliest GFP^+^ colonies appeared after 12 days of Dox induction. On day 18, 6F reprogramming generated approximately fivefold more GFP^+^ colonies than the 4F protocol (Fig. 1B).

The primary colonies were picked to establish stable iPSC lines by removal of Dox and growth in N2B27/2i/LIF (2i/LIF) medium for expansion. The 2i/LIF medium contains the MEK inhibitor PD and the GSK3 inhibitor CH and was previously shown to allow efficient derivation and maintenance of ground state pluripotency in mouse and rat ESCs 24, 32. Strikingly, stable iPSC lines could be established from 85% of the 6F primary colonies compared to only 30% of 4F colonies, indicating that 6F colonies are more advanced than 4F colonies with respect to completing reprogramming to pluripotency.

Silva et al. reported that most colonies reprogrammed from MEFs or neural stem cells using 4F were pre‐iPSCs in terms of pluripotent gene expression and X chromosome reactivation 12. The exposure of these pre‐iPS cells to 2i/LIF medium facilitates the conversion from the pre‐iPSC state to ground state pluripotency. To investigate whether the 2i/LIF medium has similar facilitating effects on 6F reprogramming in defined conditions, we repeated the above experiments and switched the N2B27/LIF medium to 2i/LIF after 8 days of Dox induction. In 6F reprogramming, all (8/8) the colonies monitored became homogenously GFP^+^ within the next 2 days (day 10) (Fig. 1C). In contrast, when the colonies were kept in N2B27/LIF with Dox, only 3 of 8 were GFP^+^, indicating that 2i/LIF medium also facilitates 6F reprogramming. However, in the 4F control, once the medium was switched to 2i/LIF on day 8, the cell clumps either died or differentiated, resulting in no iPSC colony formation (Supporting Information Table S3), again underscoring the sharp difference in reprogramming kinetics between 4F and 6F in defined conditions.

We next characterized iPSC reprogramming in N2B27 by 6F by examining the cells' degree of pluripotency. The 6F‐iPSCs expressed pluripotency markers similar to ESCs (Fig. 1D, 1E). They could be differentiated to cells representing all three somatic germ layers in vitro and in teratomas (Supporting Information Fig. S2B, S2C) and contributed to live chimeras upon blastocyst injection (Fig. 1F).

### Reprogramming MEFs to Integration‐Free iPSCs by 6F Directly in N2B27

**Figure 2:**
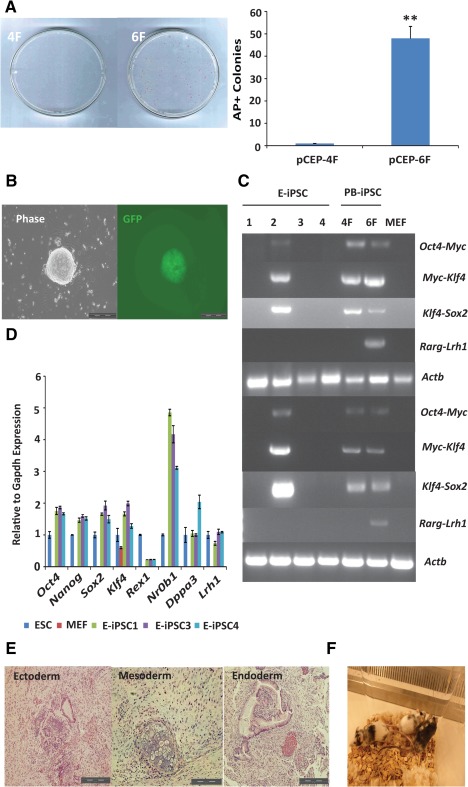
Efficient reprogramming of *Rex1‐GFP* MEFs to integration‐free iPSCs in N2B27/leukaemia inhibitory factor by 6F. **(A):** AP^+^ colonies of expressing 4F or 6F from the episomal vectors. Left panel: AP^+^ colonies. Right panel: colony numbers. **, *p* < .01. **(B):** A 6F GFP^+^ colony from the episomal vectors. Scale bar = 200 µm. **(C):** Characterization of iPSCs for loss of the episomal vectors. Whole cell lysis was used to detect residual episome or genome‐integrated episomal vectors (top four panels). *Actb* was used as a genomic DNA polymerase chain reaction (PCR) control. The bottom four panels show RT‐PCR‐amplified transcripts of exogenous factors. *Actb* expression was used as the control. E‐iPSCs were produced using episomal vectors. *PB*‐iPSCs were reprogrammed using *piggyBac* vectors. **(D):** Quantitative real‐time PCR analysis of pluripotent gene expression in iPSCs from 6F‐episomal vectors. The expression is shown relative to *Gapdh* and normalized to ESCs. **(E):** Teratomas derived from 6F E‐iPSCs. **(F):** Chimeric mice from iPSC injection into host blastocysts. Experiments were repeated at least three times, and the error bars show standard deviations from the mean of triplicate determinations of one representative experiment. Abbreviations: AP, alkaline phosphatase; ESC, embryonic stem cell; GFP, green fluorescent protein; iPSC, induced pluripotent stem cell; MEF, mouse embryonic fibroblast cell.

Integration‐free reprogramming is preferred for both basic research and potential clinical applications. Several approaches have been attempted with variable success, including those involving direct transfection of reprogramming plasmids 33, mRNA 34, nonintegrating Sendai virus 35 and Epstein‐Barr virus (EBV)‐derived episomal vectors 36. The superior reprogramming efficiency of 6F in defined conditions motivated us to investigate its application in nonintegrative reprogramming. EBV‐based episomal vectors can replicate independently of the host cell and can be gradually lost during subsequent cell divisions 37. The application of EBV episomal 4F in MEF reprogramming was, however, limited by its low efficiency 38. Therefore, we episomally expressed 6F in *Rex1‐GFP* MEFs by transfecting 1.0 × 10^6^ cells with either pCEP‐4F or pCEP‐4F plus pCEP‐2F (the transfection efficiency was about 30%) (Supporting Information Fig. S1). Morphological changes with epithelial‐like cells and tiny colonies were evident on day 8. Approximately 50–60 AP^+^ colonies could usually be obtained on day 18 (Fig. 2A). Importantly, almost all the primary colonies could be established as stable iPSC lines in 2i/LIF (Fig. 2B). By contrast, in the 4F control, morphological changes could only be detected on day 10, and on average, only one GFP^+^ colony was obtained per transfection. Whole cell lysis DNA PCR revealed that 82% (18/22) of the lines lost the episomal vectors at passage 10 (Fig. 2C). These integration‐free iPSCs expressed pluripotency markers (Fig. 2D), formed mature teratomas, and contributed to live chimeric mice (Fig. 2E, 2F). The high efficiency of the episomal 6F system was highly reproducible in various mouse strains, including C57BL/6J. Therefore, the chemically defined episomal 6F reprogramming system provides a robust and very simple approach to produce integration‐free mouse naïve iPSCs.

### Activation of RA Signalling by Retinoids Enhances Reprogramming of MEFs to iPSCs

**Figure 3:**
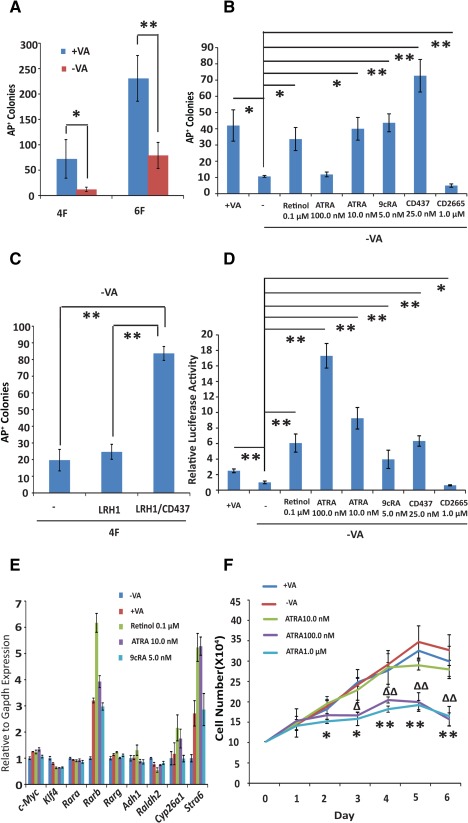
Retinoic acid (RA) signalling promotes mouse embryonic fibroblast cell (MEF) reprogramming in the defined condition. **(A):** The effect of withdrawing VA (retinol) from N2B27/leukemia inhibitory factor (LIF) on 4F and 6F reprogramming. *, *p* < .05; **, *p* < .01. **(B):** Reprogramming MEFs in medium containing retinoids. *, *p* < .05; **, *p* < .01. **(C):** The retinoic acid receptor gamma agonist CD437 (25.0 nM) increases AP^+^ colony numbers by 4F plus LRH1 in −VA medium. **(D):** RA signalling activated by various retinoids in a luciferase assay. **(E):** Quantitative real‐time PCR analysis of gene expression in MEFs cultured in N2B27/LIF in the presence of retinoids for 2 days. These genes include pluripotency‐related genes (*c‐Myc*, *Klf4*), *Rar*s (α, β, γ) and genes involved in retinol metabolism (*Adh1*, *Raldh2*, *Cyp26a1*, and *Stra6*). The expression levels are shown relative to *Gapdh* and normalized to embryonic stem cells. **(F):** Proliferation of MEFs in retinoids. MEFs (1 × 10^5^) were plated in N2B27/LIF with or without vitamin A or ATRA. Cells were counted daily for 6 days. Δ, *p* < .05; ΔΔ, *p* < .01: ATRA 100.0 nM compared with −VA; *, *p* < .05; **, *p* < .01: ATRA 1.0 µM compared with −VA. In the MEF reprogramming experiments, AP^+^ colonies were scored on day 18 after transfection. The experiments were repeated at least three times, and the error bars shown standard deviations from the mean of triplicate determinations in one representative experiment. Abbreviations: AP, alkaline phosphatase; ATRA, all‐*trans* retinoic acid; 9cRA, 9‐*cis*‐retinoic acid; LRH1, liver receptor homolog‐1; VA, vitamin A.

The establishment of a chemically defined reprogramming condition allowed us to dissect the roles of RA signalling in reprogramming in a controlled context. Coincidentally, retinol acetate, the acetyl form of VA, is one of the key components in the B27 supplement in N2B27/LIF medium 27. This fact allows us to directly study the effect of retinol and its metabolites in this medium. We began by comparing the reprogramming efficiency in N2B27/LIF with or without supplementing VA. We transfected MEFs with 4F or 6F expression *PB* vectors and plated them in either the standard N2B27/LIF medium (media with VA referred to as +VA) or N2B27/LIF without VA (referred to as −VA, made from commercially available B27 without VA), in feeder‐free conditions. However, the exact VA concentration in the commercially available B27 supplement is not publicly available. The −VA medium decreased AP^+^ colony numbers by up to sevenfold and approximately two‐ to threefold in 4F and 6F reprogramming, respectively (Fig. 3A). Supplementation with other retinoids, including retinol, ATRA (10.0 nM), 9cRA, and the RARG agonist CD437, individually in −VA medium restored or even further improved reprogramming efficiency to the level of standard N2B27 or +VA medium (Fig. 3B). Conversely, the presence of the RAR antagonist CD2665 further reduced the numbers of AP^+^ colonies in −VA medium (Fig. 3B). The positive effect of VA in reprogramming initially appears counterintuitive as RA is frequently used to induce differentiation in vitro. However, it is important to note that the concentrations of retinoids used in this experiment (ATRA, 10.0 nM) were within the physiological range (1.0–10.0 nM) 39 and much lower than the differentiation induction level (ATRA, 100.0 nM–1.0 µM). Indeed, ATRA at 100 nM was deleterious to reprogramming (Fig. 3B).

We then asked if RARG agonists, such as retinoids or CD437 alone, could recapitulate the positive effect of RARG overexpression in 6F reprogramming in MEFs. We found that the addition of CD437 also produced three‐ to fourfold more colonies (Fig. 3C) than either 4F/LRH1 or 4F alone. Despite this fact, neither CD437 nor other retinoids significantly altered the reprogramming kinetics, as the time point of emergence of the GFP^+^ cells in *Rex1‐GFP* MEF reprogramming is similar to that of cells undergoing 4F reprogramming. To confirm that the retinoids were able to activate RA signalling in MEFs, we performed a luciferase assay using a reporter with the RARE 40. As expected, the addition of retinoids substantially activated luciferase activities, while CD2665 further dampened the activities compared to the no VA control (Fig. 3D). Exogenous retinoids also increased the expression of known downstream target genes of the RA signalling pathway, including *Rarb* and *Cyp26a1* (Fig. 3E). ATRA at 100.0 nM led to much higher activity in the luciferase assay (Fig. 3D) but was nevertheless deleterious to MEF reprogramming (Fig. 3B).

It has been previously shown that increased cell proliferation promotes MEF reprogramming 41 and that retinoids can affect cell proliferation in a dose‐dependent manner 42, 43. We thus asked if the positive effect of retinoids on reprogramming could be attributed to an alteration of cell proliferation. However, neither +VA nor ATRA at 10.0 nM noticeably altered the proliferation of MEFs (Fig. 3F). High concentrations of ATRA (100.0 nM and 1.0 µM) were in fact detrimental (Fig. 3F). Therefore, the effect of RA signalling on reprogramming does not appear to be cell cycle independent. RA signalling likely promotes MEF reprogramming through alternative mechanisms, such as regulation of the *Oct4* locus 13, 44, 45, 46.

### Ligand‐Independent Function of RARs in Reprogramming

**Figure 4:**
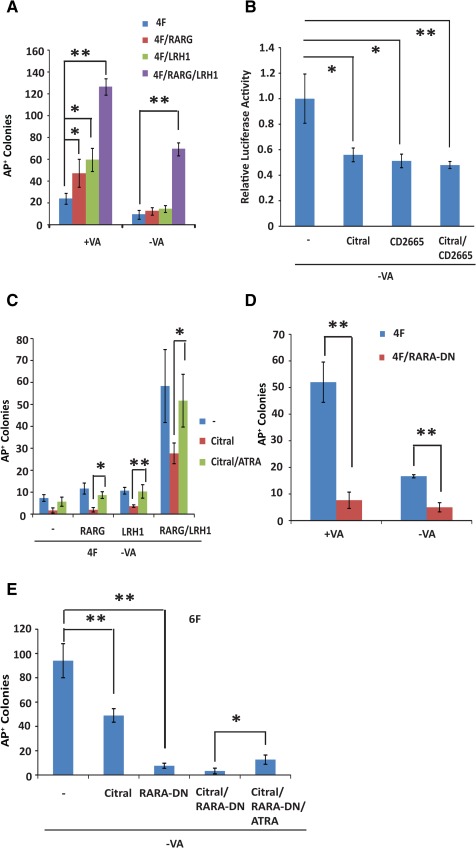
Reprogramming mouse embryonic fibroblast cells (MEFs) by 6F is both retinoic acid (RA) receptor ligand‐dependent and ligand‐independent. **(A):** MEFs were reprogrammed by 4F alone, 4F plus either RARG or LRH1, or 4F plus both RARG and LRH1 (6F) in +VA and −VA medium. Significantly more AP^+^ colonies are obtained by 6F, even in −VA. **(B):** Blocking RA signalling by citral (50.0 μM) or CD2665 (1.0 μM) as measured in a luciferase assay. **(C):** Blocking 4F‐ or 6F‐mediated MEF reprogramming by citral (50.0 μM). Adding ATRA rescues reprogramming. Note that 6F produces substantially more AP^+^ colonies in the presence of citral compared with the other conditions. **(D):** The blocking of MEF reprogramming by RARA‐DN. **(E):** The effects of blocking RA signalling by either citral (50.0 μM) or RARA‐DN on MEF reprogramming. Note that adding citral and expressing RARA‐DN almost completely blocked reprogramming. Adding ATRA at 10.0 nM could partially rescue the block. In the MEF reprogramming experiments, AP^+^ colonies were scored on day 18 after transfection. *, *p* < .05; **, *p* < .01. The experiments were repeated at least three times, and the error bars show standard deviations from the mean of triplicate determinations in one representative experiment. Abbreviations: AP, alkaline phosphatase; ATRA, all‐*trans* retinoic acid; LRH1, liver receptor homolog‐1; RARA‐DN, dominant‐negative form of RARA; RARG, retinoic acid receptor gamma; VA, vitamin A.

We observed in the previous experiments that the removal of VA from N2B27/LIF drastically reduced the colony number in 4F reprogramming, but only a relatively mild colony number reduction was observed in 6F reprogramming (Fig. 3A). Moreover, supplementation of CD437 did not change the kinetics of *Rex1* reactivation in 4F reprogramming, unlike the early *Rex1* reactivation observed in 6F reprogramming. These results indicated that some of the positive effects of RARG and LRH1 on reprogramming must be mediated by a RA‐ or RAR ligand‐independent mechanism. To further dissect these possibilities, we expressed 4F using the constitutive CAG promoter but controlled *Rarg* expression in a Dox‐inducible manner. The expression of exogenous RARG increased AP^+^ colony numbers two‐ to threefold across all the tested Dox induction concentrations (Fig. 4A and Supporting Information Fig. S3A), indicating that VA in the standard N2B27 likely limits further increases in RA signalling. Moreover, in −VA medium, exogenous RARG was no longer able to boost MEF reprogramming (Fig. 4A and Supporting Information Fig. S3A). Therefore, the effect of expressing RARG alone (no endogenous LRH1 in MEFs) is primarily dependent on VA or its metabolites, that is, ligand‐dependent. Similarly, the promoting effect of RARA alone on reprogramming was also VA‐dependent (Supporting Information Fig. S3B). Interestingly, expressing LRH1 alone also promoted 4F reprogramming in +VA medium (Fig. 4A and Supporting Information Fig. S3B), as shown in previous reports using serum‐containing media 46. However, again the effect was largely absent when −VA medium was used (Fig. 4A and Supporting Information Fig. S3B). Therefore, LRH1 promotes reprogramming likely through interaction with the relatively low levels of endogenous RARs in MEFs. At these levels of RARs, the effects of exogenous LRH1 appear to be primarily dependent on the RAR ligands.

To ensure that our interpretation was not confounded by endogenous RA signalling activation, we blocked endogenous RA production using citral, a chemical inhibitor of retinol oxidation 47, 48. The addition of citral in −VA medium further reduced the luciferase activities in MEFs, similar to CD2665 (Fig. 4B), confirming its function in blocking endogenous RA synthesis. Citral and CD2665 together, however, did not show an obvious additive effect on luciferase activities, indicating effective blocking of RA signalling by either approach. In MEF reprogramming, the addition of citral in −VA medium caused further reduction of AP^+^ colonies by two‐ to threefold when reprogrammed by 4F alone, 4F plus RARG, or 4F plus LRH1 (Fig. 4C), which could be rescued by adding ATRA (10.0 nM) (Fig. 4C). This result further confirmed that expressing LRH1 and RARG individually in MEF reprogramming is dependent on RAR ligands. Citral also impeded 6F reprogramming in −VA medium, which could also be rescued by ATRA (Fig. 4C). However, even when both endogenous RA synthesis and exogenous RA supplement were absent (citral in −VA medium), there were still considerably more AP^+^ colonies generated by 6F than by 4F, or by 4F with either RARG or LRH1 (Fig. 4A, 4C). Therefore, the synergistic effect of exogenous RARG and LRH1 on reprogramming can be mediated by a ligand‐independent action as well as a ligand‐dependent one.

To further corroborate our findings, we blocked RA signalling by expressing a RARA‐DN 49. MEFs express *R*ars, in particular, *Rarb* (Supporting Information Fig. S4). We previously found that RARA‐DN almost completely blocked MEF reprogramming in serum‐containing medium 13. In both +VA and −VA medium, expressing RARA‐DN essentially shut down RA signalling as measured by the luciferase assay (Supporting Information Fig. S5A). RARA‐DN expression also drastically reduced AP^+^ colony numbers by both 4F and 6F to a similar degree, thus completely negating the facilitating effect of RARG and LRH1 (Fig. 4D, 4E). The surviving AP^+^ colonies, as expected, did not appear to express RARA‐DN (Supporting Information Fig. S5B). Adding citral to the RARA‐DN‐expressing cells further reduced the colony number. Conversely, adding back ATRA could not effectively rescue the blockage by RARA‐DN (Fig. 4E). Therefore, RARA‐DN effectively inactivated both ligand‐dependent and ligand‐independent actions of RARs, confirming the essential role of RARs in reprogramming.

### RA Signalling Promotes Reprogramming EpiSCs to Ground State Pluripotency

Having established the roles of RARs in MEF reprogramming, we expanded our scope of investigation to study whether RA signalling is important in EpiSC reprogramming. EpiSCs are pluripotent stem cells distinct from ground state ESCs with respect to morphology, signalling dependence, gene expression, and epigenetic profiles 50. They are derived from postimplantation epiblasts and are cultured in N2B27 medium containing AF and are not permissible in the 2i/LIF condition. They can be reprogrammed to ground state pluripotency by exogenous factors such as KLF4, LRH1, and NANOG 25, 51, 52.

**Figure 5:**
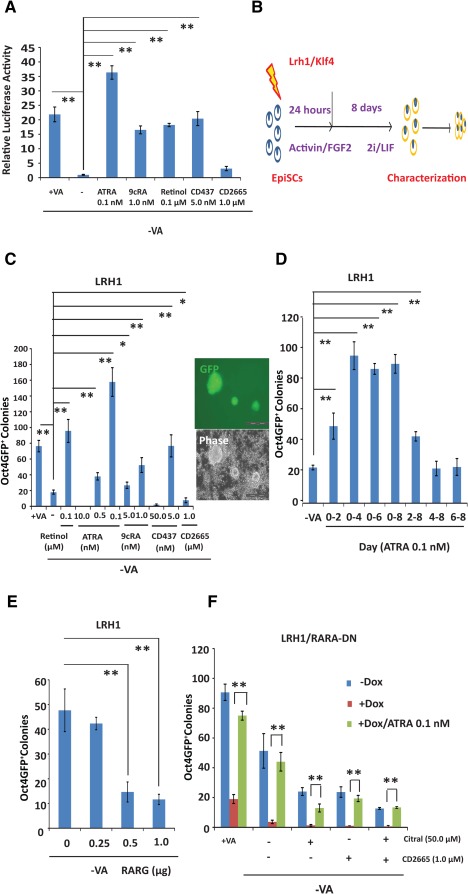
Retinoic acid (RA) signalling in EpiSC reprogramming. **(A):** RA signalling as measured by a luciferase assay in EpiSCs cultured in N2B27/activin/FGF2 with VA and other retinoids. The activities are shown relative to that of Renilla luciferase and normalized to that of EpiSCs in −VA. **(B):** A diagram of EpiSC reprogramming in 2i/LIF. **(C):** The effects of retinoids on reprogramming *Oct4‐GFP* reporter EpiSCs to induced pluripotent stem cells by LRH1. *, *p* < .05; **, *p* < .01. Scale bar = 200 µm. **(D):** RA signalling at various stages of EpiSC reprogramming. **, *p* < .01. **(E):** The effects of expressing RARG (under the CMV early enhancer/chicken β actin promoter) on reprogramming EpiSCs by LRH1. Note that even small amounts of *Rarg PB* transposon have deleterious effects. *, *p* < .05; **, *p* < .01. **(F):** Blocking LRH1‐mediated EpiSC reprogramming by ligand depletion (citral), RA receptor inhibition (CD2665) or expression of RARA‐DN. Reprogramming was partially restored by 0.1 nM ATRA. Oct4‐GFP^+^ colonies were scored on day 9 after transfection. *, *p* < .05; **, *p* < .01. In all the experiments, Oct4‐GFP^+^ colonies were scored on day 9 after transfection. The experiments were repeated at least three times, and the error bars show standard deviations from the mean of triplicate determinations in one representative experiment. Abbreviations: ATRA, all‐*trans* retinoic acid; 9cRA, 9‐*cis*‐retinoic acid; EpiSCs, epiblast stem cells; FGF2, fibroblast growth factor 2; LIF, leukemia inhibitory factor; LRH1, liver receptor homolog‐1; RARA‐DN, dominant‐negative form of RARA; RARG, retinoic acid receptor gamma; VA, vitamin A.

EpiSCs expressed much lower levels of RARs than MEFs (Supporting Information Fig. S4). Nevertheless, retinoids, such as retinol, ATRA, 9cRA, and CD437, substantially increased RARE luciferase reporter activities in EpiSC, and removing VA from N2B27 considerably decreased the activities (Fig. 5A). Remarkably, a much lower concentration of retinoids was needed to induce robust luciferase activities in EpiSCs. For example, 10.0 nM ATRA in MEFs efficiently activated the reporter, whereas 0.1 nM was able to robustly activate the reporter in EpiSCs. Importantly, retinoids at these low concentrations in EpiSCs did not substantially change the expression of most key genes involved in pluripotency or differentiation (Supporting Information Fig. S6A).

**Figure 6:**
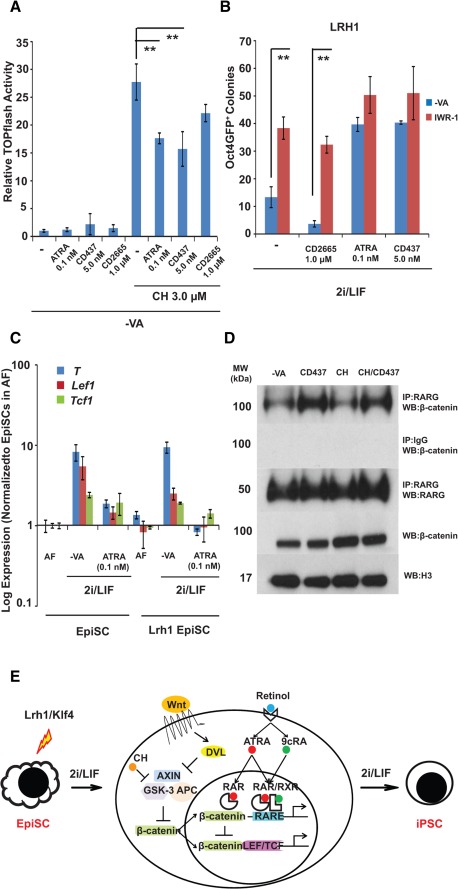
Retinoic acid (RA) signalling modulates the Wnt pathway. **(A):** Wnt signalling as measured by a TOPflash assay. EpiSCs were cultured in N2B27‐VA or supplemented with ATRA, CD437, or CD2665 with or without CH for 24 hours before the assay. The activities are shown relative to Renilla luciferase and normalized to EpiSC in N2B27‐VA. **, *p* < .01. **(B):** The attenuation of Wnt signalling by IWR‐1 improves EpiSC reprogramming. After *Lrh1* transfection, *Oct4‐GFP*EpiSCs were cultured in 2i/LIF medium without VA and supplemented with ATRA, CD437, or CD2665 with or without IWR‐1 (2.0 µM) for 4 days. The cells were subsequently cultured in 2i/LIF‐VA for another 4 days. **, *p* < .01. **(C):** The activation of RA signalling downregulates CH‐induced expression of Wnt target genes. EpiSCs or *Lrh1* transfected EpiSCs were cultured in 2i/LIF‐VA and 2i/LIF‐VA supplemented with ATRA for 24 hours for quantitative real‐time PCR analysis. The expression is shown relative to *Gapdh* and normalized to EpiSCs in N2B27/AF‐VA. **(D):** The interaction of RARG and β‐catenin. Nuclear extracts of EpiSCs cultured in N2B27‐VA with CD437 (5.0 nM) or CH (3.0 µM) were immunoprecipitated with an anti‐RARG antibody. The pull‐down proteins were blotted with an anti‐β‐catenin antibody and an anti‐RARG antibody, sequentially. Rabbit IgG was the negative control, and Histone H3 was the loading control. Western blotting of β‐catenin in EpiSCs prior to immunoprecipitation was also performed. In all experiments, iPSC colonies were scored on day 9 after transfection. The experiments were repeated at least three times, and the error bars show standard deviations from the mean of triplicate determinations in one representative experiment. **(E):** A proposed model of the interaction between Wnt and RA signalling in EpiSCs reprogramming. Abbreviations: AF, activin/fibroblast growth factor 2; APC, Adenomatosis Polyposis Coli; ATRA, all‐*trans* retinoic acid; CH, CHIR9902; 9cRA, 9‐*cis*‐retinoic acid; DVL, dishevelled; EpiSCs, epiblast stem cells; GSK, glycogen synthase kinase; iPSC, induced pluripotent stem cell; IP, immunoprecipitation; LEF, lymphoid enhancing factor; LIF, leukemia inhibitory factor; LRH1, liver receptor homolog‐1; MW, molecular weight; RAR, RA receptor; RARE, retinoic acid response element; RARG, retinoic acid receptor gamma; RXR, retinoid X receptor; TCF, T‐cell factor; VA, vitamin A; WB, Western blot.

We next attempted to reprogram *Oct4‐GFP* EpiSCs 25 to iPSCs by expression of exogenous LRH1 (Fig. 5B). The reprogramming efficiency of EpiSCs could be easily determined by monitoring Oct4‐GFP^+^ iPSC colonies 25. The medium was switched to 2i/LIF supplemented with various retinoids after recovery in AF. Consistent with the luciferase assay results, low concentrations of retinoids, particularly ATRA (0.1 nM), substantially increased iPSC colonies (Fig. 5C), whereas high concentrations of retinoids or the RAR antagonist CD2665 were deleterious to reprogramming (Fig. 5C). The iPSCs from EpiSCs expressed comparable levels of key pluripotency genes as ESCs (Supporting Information Fig. S6B). The positive effect of RA was also evident in KLF4‐mediated EpiSC reprogramming (Supporting Information Fig. S6D) 25. Therefore, RA signalling also functions in EpiSC reprogramming and is highly dose‐sensitive.

We attempted to delineate the temporal window during which RA signalling activation is required in LRH1‐mediated EpiSCs reprogramming. We found that adding ATRA (0.1 nM) during the first 2–4 days produced two‐ to fivefold more iPSC colonies, whereas continuous ATRA treatment did not further increase iPSC colony number (Fig. 5D and Supporting Information Fig. S6C). Thus, RA signalling activation in early phases of EpiSC reprogramming appears to be important.

In addition to retinoids, we also attempted to activate RA signalling by expressing exogenous RARs in EpiSC reprogramming. Surprisingly, in LRH1‐mediated EpiSC reprogramming, the coexpression of RARG actually reduced reprogramming efficiency in EpiSCs (Fig. 5E). High levels of RA signalling by either RARG overexpression or high concentrations of retinoids are thus detrimental in EpiSC reprogramming, which is again highly sensitive to the RA dose. Conversely, the expression of RARA‐DN severely decreased EpiSC reprogramming by both exogenous LRH1 and KLF4 (Fig. 5F and Supporting Information Fig. S6E). Furthermore, the addition of citral or CD2665 and the coexpression of RARA‐DN completely abolished EpiSC reprogramming (Fig. 5F), revealing that RA signalling is indispensable in EpiSC reprogramming to ground state pluripotency. Similar to MEF reprogramming (Fig. 4E), in both LRH1 and KLF4‐mediated EpiSC reprogramming, exogenous ATRA at 0.1 nM only partially rescued the inhibition by RARA‐DN (Fig. 5F and Supporting Information Fig. S6D).

### RA Signalling Negatively Regulates Wnt Signalling Through Direct Interaction of RARG and β‐Catenin

We next investigated the potential mechanism of RA signalling in EpiSC reprogramming given that compared to MEF reprogramming, this process likely requires relatively few epigenetic changes. We noted that EpiSCs were reprogrammed to iPSCs in 2i/LIF medium, which contains PD and CH. A major consequence of GSK3 inhibition by CH is stabilization of β‐catenin, which increases canonical Wnt signalling 53. In mouse ESCs, the effective concentration of CH has been empirically determined as 3.0 µM, which causes partial inhibition of GSK3 24. The increased levels of β‐catenin function primarily to derepress pluripotency genes mediated by TCF3, including *Nanog*, *Klf2*, and *Esrrb* 54, 55, 56. However, excessive canonical Wnt activities in rat ESCs can induce Wnt target genes, such as *Cdx1* and *T*, affecting nuclear β‐catenin‐binding lymphoid enhancing factor 1 (LEF‐1) and causing heterogeneity in rat ESC culture 57. We speculated that fine‐tuning Wnt signalling could also be important in EpiSC reprogramming. Increased CH caused higher β‐catenin‐mediated activity in the TOPflash reporter assay 58 in EpiSCs (Supporting Information Fig. S7A), and higher CH concentrations were deleterious in LRH1‐mediated EpiSC reprogramming as measured by Oct4GFP^+^ iPSC colony numbers (Supporting Information Fig. S7B). In both breast and colon cancer cells, RA decreases the activity of the β‐catenin‐LEF/TCF signalling pathway via direct interaction of RARs and β‐catenin 59. In view of this fact, we asked if there is crosstalk between RA signalling and Wnt signalling in EpiSC reprogramming. We repeated TOPflash experiments in EpiSCs in the presence of RAR agonists or an antagonist. The agonists, ATRA and CD437, but not the antagonist CD2665, significantly reduced TOPflash activities (Fig. 6A), showing that RA signalling likely negatively regulates β‐catenin activities. To further address this possibility, we tested the effects of IWR1, a chemical stabilizer of the AXIN2 protein that degrades β‐catenin to inhibit Wnt signalling 60, in LRH1‐mediated EpiSC reprogramming. IWR‐1 significantly increased iPSC colony numbers in the absence of VA, but no added effect on reprogramming was observed when either ATRA or CD437 was present in the medium (Fig. 6B). Therefore, concurrent inhibition of Wnt signalling by IWR‐1 and activation of RA signalling by retinoids did not further improve reprogramming. Conversely, IWR‐1 rescued the reprogramming blockage by CD2665 (Fig. 6B). Moreover, in the presence of RA, among the Wnt target genes examined, *Lef‐1* and *T* were substantially downregulated in both wild‐type EpiSCs and those undergoing LRH1‐mediated reprogramming (Fig. 6C). These results thus suggest that β‐catenin/canonical Wnt signalling might act downstream of RA signalling in EpiSC reprogramming.

We subsequently investigated the possibility that RARs and β‐catenin might directly interact in EpiSCs and performed coimmunoprecipitation using an antibody to RARG. The pull‐down complexes were probed with a β‐catenin antibody. In the absence of CD437, there was weak interaction between RARG and β‐catenin (Fig. 6D). Once CD437 was added, β‐catenin was substantially enriched in the RARG pull‐down complexes (Fig. 6D), confirming a ligand‐dependent regulation of β‐catenin's availability and thus of Wnt signalling by RARG. We conclude that one mechanism of RA signalling promoting EpiSC reprogramming is modulating or negatively regulating Wnt signalling through direct RAR‐β‐catenin physical interaction.

## Discussion

Reprogramming in serum‐containing medium and on feeder cells can complicate the efforts to unravel the mechanistic details of the reprogramming process 61. Reprogramming MEFs in defined medium usually requires expression of reprogramming factors by integrative retroviruses, which are prone to leakiness and reactivation of reprogramming factors in iPSCs 62. We previously reported that the addition of two transcription factors, LRH1 and RARG, substantially increases the efficiency of reprogramming and shortens its course 13. In this study, we tested the feasibility of directly reprogramming MEFs into iPSCs using 6F in chemically defined N2B27/LIF medium. We further demonstrated that 6F‐episomal reprogramming provides a simple and robust approach for the derivation of integration‐free iPSCs. The high efficiency of 6F reprogramming in defined conditions permits us to investigate the role of RA signalling in reprogramming.

Retinol acetate, or vitamin A, is a component of B27 supplement in N2B27 medium and the precursor of retinoic acid. We showed that removing VA from the medium, adding a RAR antagonist, or inhibiting endogenous RA synthesis by citral markedly impeded MEF reprogramming in both 4F and 6F systems, whereas activation of RA signalling by supplementation of retinoids or RAR agonist improved reprogramming. These results are in accordance with those of Hou et al., who recently reported that the use of the synthetic retinoic acid receptor ligand TTNPB significantly enhanced MEF reprogramming 10. We also determined that the dosage requirement for RA signalling is highly cell type‐specific. For example, 10.0 nM ATRA promotes reprogramming of MEFs, whereas EpiSC reprogramming only needs 0.1 nM ATRA. These ATRA concentrations are 10–100‐fold and 1,000–10,000‐fold lower than those used in ESC differentiation (0.1–1.0 µM), respectively 63, 64, 65. Therefore, reprogramming somatic cells to ground state pluripotent stem cells operates in a narrow range of RA concentrations. The fact that only very low concentrations of RA are needed for EpiSCs reprogramming accounts for the inhibitory effect of expressing exogenous RARs, as the copy number of an expression cassette integrated in the genome cannot be lower than one. It will be interesting to investigate whether exogenous RARs can promote EpiSCs reprogramming efficiency when endogenous RARs are inactivated.

In addition to depending on RA, exogenous RARG, when coexpressed with LRH1, can operate in a RA‐independent manner in reprogramming MEFs. RARs appear to exhibit similar mechanisms in other cells types. For example, in F9 embryonal carcinoma stem cells, RARA regulates the expression of certain genes independently of the RA ligand by modulating the CpG methylation status of specific promoter regions 66. Moreover, RARA interacts with estrogen receptor alpha (ERα) in breast cancer cells and co‐occupies regulatory regions in an ER‐dependent manner 67, 68. Further investigation is needed to understand the molecular mechanisms whereby RARs partner with LRH1 and/or other pluripotent factors to facilitate reprogramming independently of their ligands. One potential approach is to perform genome‐wide mapping of the cobinding sites of RARs and their partners during reprogramming.

RA signalling also promoted EpiSC reprogramming. The regulation of β‐catenin at physiological levels has been shown to be essential in cell fusion reprogramming 69, but the excess inhibition of GSK3 by CH shown in this study was deleterious in EpiSC reprogramming, as dampening β‐catenin activity using the AXIN2 stabilizer IWR‐1 facilitated reprogramming. We demonstrated that the activation of RARG by its ligands attenuated the activity of β‐catenin through direct physical interaction, which improves Lrh1‐ or Klf4‐mediated EpiSCs reprogramming (Fig 6E). Our chemically defined condition for MEF reprogramming in this study does not include exogenous Wnt modulators. However, multiple lines of evidence support the idea that Wnt signalling promotes somatic cell reprogramming in a stage‐specific manner 69, 70, 71, 72, 73. It will therefore be interesting to characterize the interaction of RA and Wnt signalling in MEF reprogramming, which will extend our knowledge of RA's established function in facilitating the reactivation of key pluripotency loci, such as *Oct4*.

## Conclusion

In this study, we show that RA signalling plays essential role in 4F and 6F mediated MEF reprogramming in both ligand dependent and independent manner. Moreover, mild activation of RA signalling also facilitates reprogramming epiblast stem cells into naïve pluripotent stem cells by modulating Wnt signalling. Altogether, the results demonstrate the importance of RA signalling in cellular reprogramming. It will be of great interest to explore the function and mechanism of RA signalling in acquiring human naïve pluripotency through reprogramming.

## Author Contributions

J.Y.: conception and design, collection and assembly of data, data analysis and interpretation, manuscript writing; W.W. and J.O.: provision of study material, data analysis and interpretation; L.C.: data analysis and interpretation; L.L.: experimental design, data analysis, interpretation and manuscript writing; P.L.: conception and design, financial support, data analysis and interpretation, manuscript writing, final approval of the manuscript.

## Disclosure of Potential Conflict of Interest

The authors declare no competing financial interests.

## Supporting information

Supplementary Figure S1

Supplementary Figure S2

Supplementary Figure S3

Supplementary Figure S4

Supplementary Figure S5

Supplementary Figure S6

Supplementary Figure S7

Supplementary Table S1

Supplementary Table S2

Supplementary Table S3
