# Supplementary figures and images for: Signalling Through Retinoic Acid Receptors is Required for Reprogramming of Both Mouse Embryonic Fibroblast Cells and Epiblast Stem Cells to Induced Pluripotent Stem Cells
Source: Stem Cells. 2015 Apr 23;33(5):1390–404. doi: 10.1002/stem.1926 (PMC4863141; doi:10.1002/stem.1926)

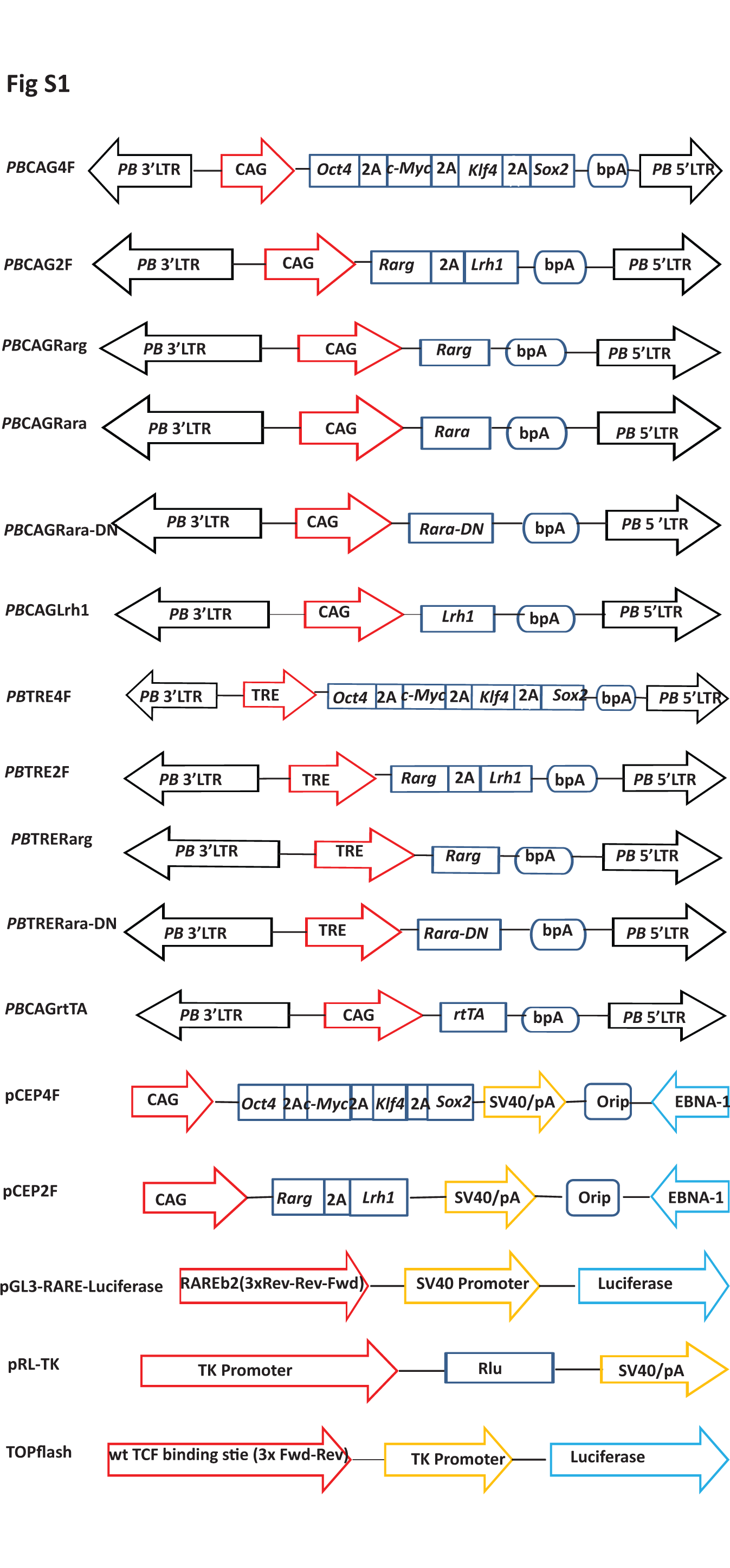

Supplement: Supplementary file 1 — Supplementary Figure S1 [file STEM-33-1390-s001.tif]

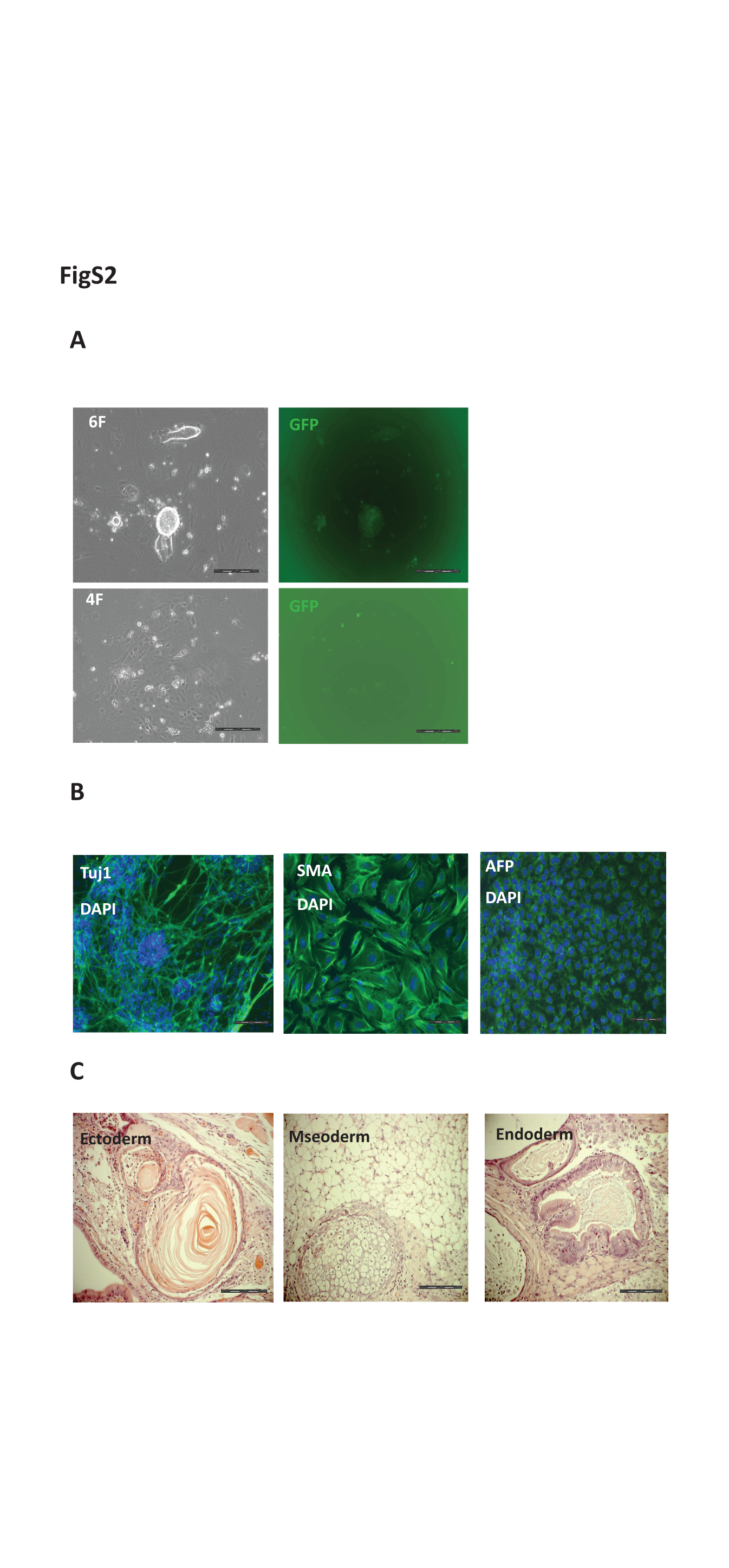

Supplement: Supplementary file 2 — Supplementary Figure S2 [file STEM-33-1390-s002.tif]

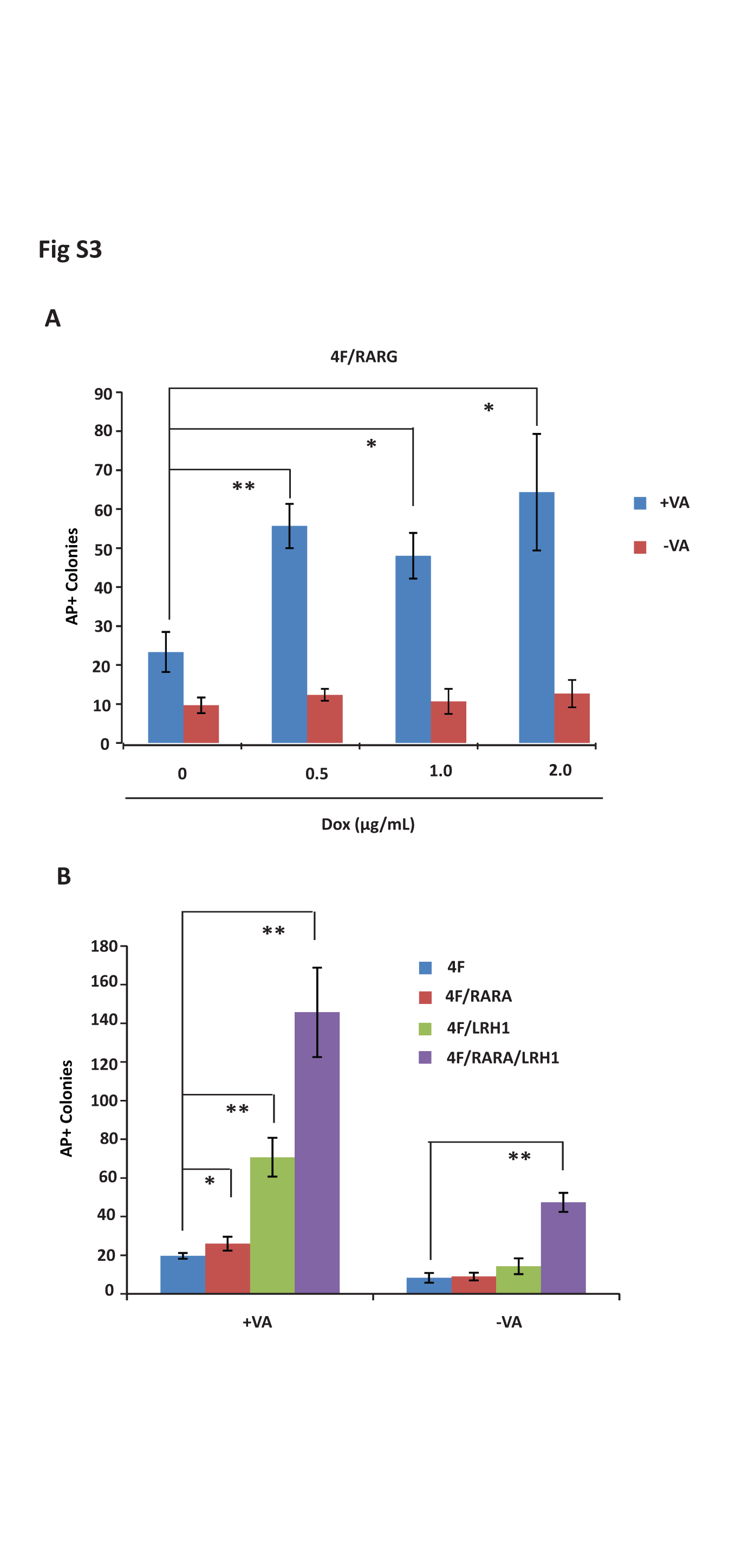

Supplement: Supplementary file 3 — Supplementary Figure S3 [file STEM-33-1390-s003.tif]

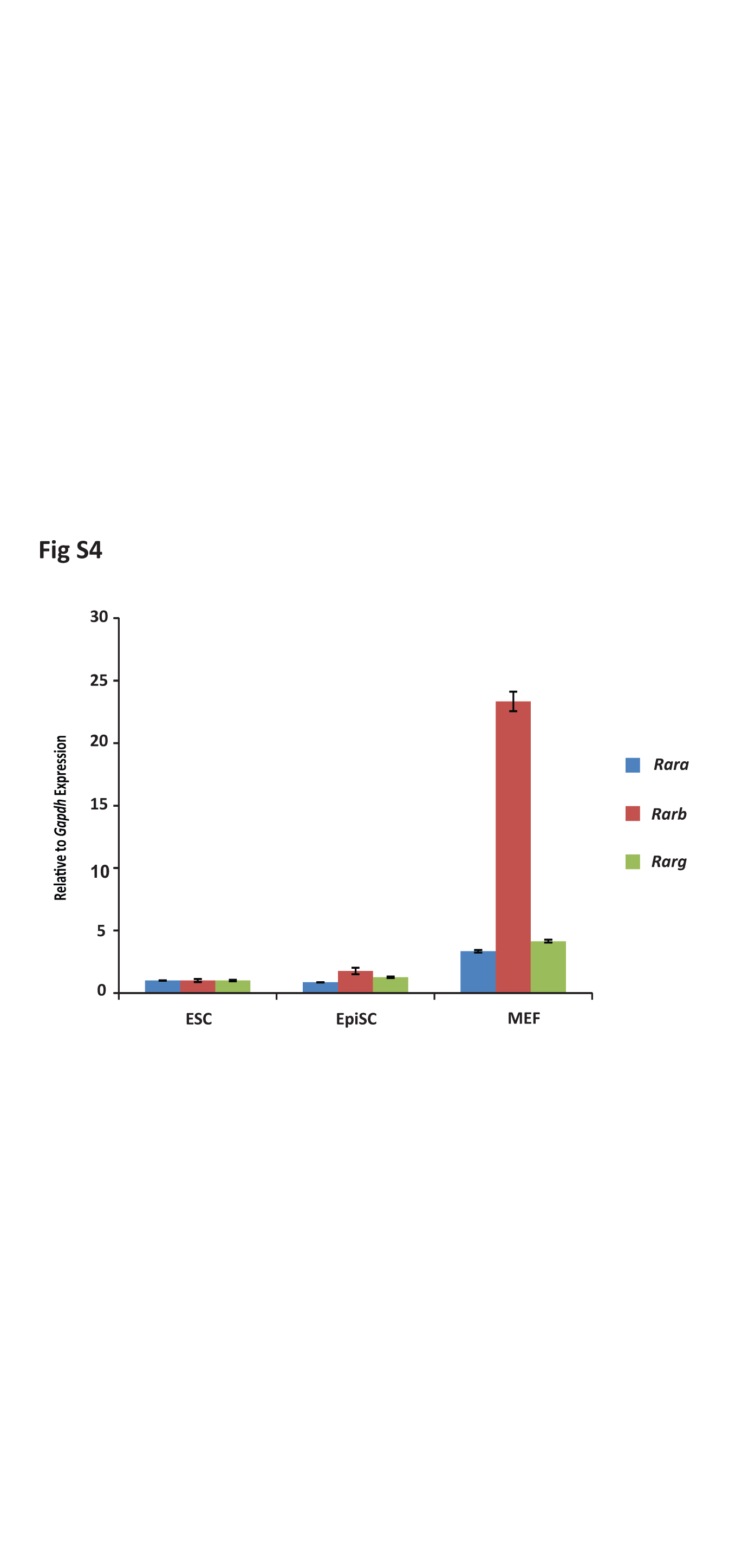

Supplement: Supplementary file 4 — Supplementary Figure S4 [file STEM-33-1390-s004.tif]

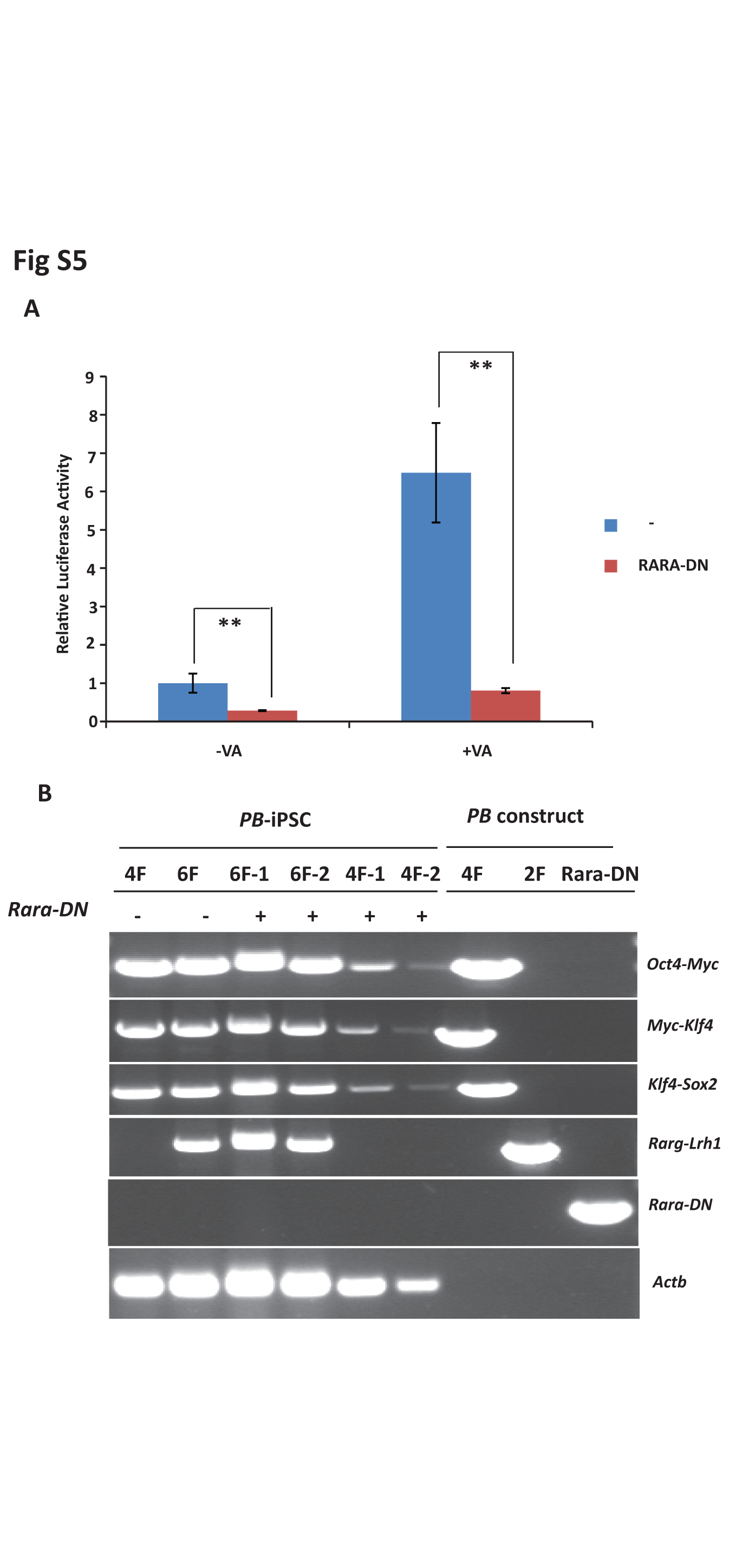

Supplement: Supplementary file 5 — Supplementary Figure S5 [file STEM-33-1390-s005.tif]

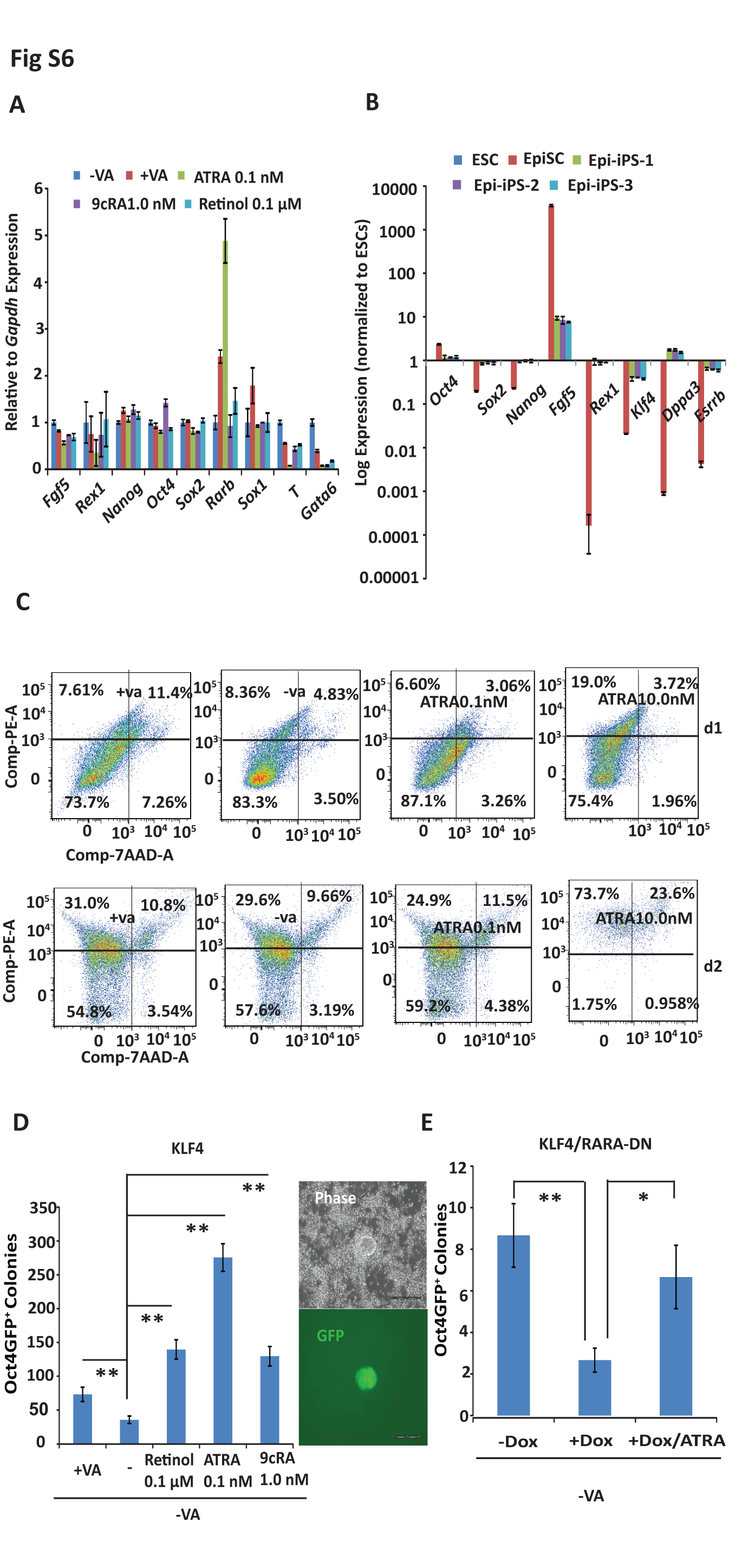

Supplement: Supplementary file 6 — Supplementary Figure S6 [file STEM-33-1390-s006.tif]

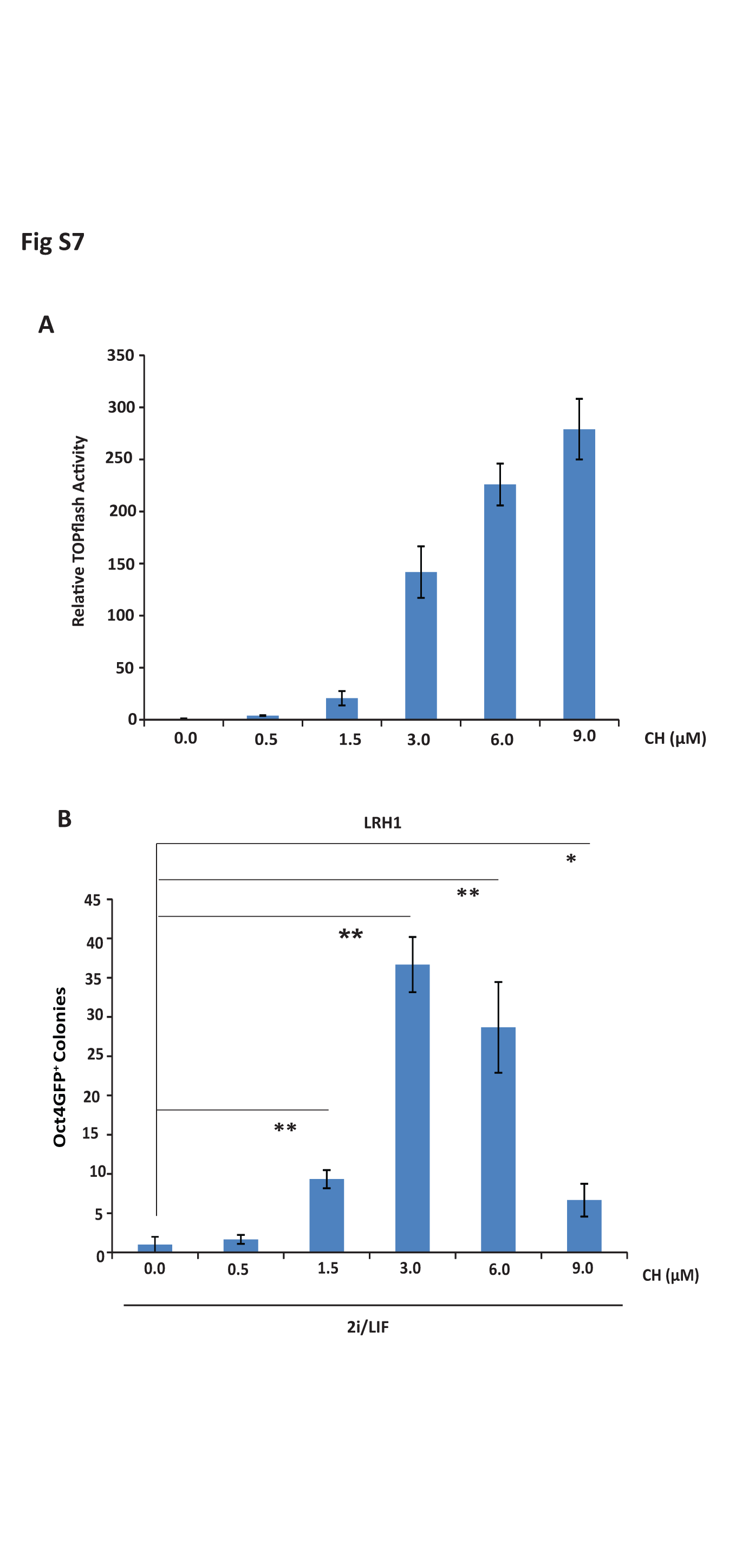

Supplement: Supplementary file 7 — Supplementary Figure S7 [file STEM-33-1390-s007.tif]
