# Supplementary material for: Signalling Through Retinoic Acid Receptors is Required for Reprogramming of Both Mouse Embryonic Fibroblast Cells and Epiblast Stem Cells to Induced Pluripotent Stem Cells
Source: Stem Cells. 2015 Apr 23;33(5):1390–404. doi: 10.1002/stem.1926 (PMC4863141; doi:10.1002/stem.1926)
Supplement: Supplementary file 8 — Supplementary Table S1 [file STEM-33-1390-s008.docx]

| Name | Sequence |
| --- | --- |
| *Oct4-c-Myc* junction | Forward: 5’ GCCCCCAGGTCCCCACTTTG 3’  Reverse: 5’ CCAGCTGATCGGCGGTGGAG 3’ |
| *c-Myc-Klf4* junction | Forward: 5’ CAGATCAGCAACAACCGCAAGTG 3’  Reverse: 5’ GCTGCTCGCCGGGGAAGAC 3’ |
| *Klf4-Sox2* junction | Forward: 5’ ACTATGCAGGCTGTGGCAA 3’  Reverse: 5’ TTGCTGCGGGCCCGGCGGCT 3’ |
| *Rarg-Lrh1* junction | Forward: 5’ TGCTGGAAGCCCTGAGGCTCTAT 3’  Reverse: 5’ CAGTAGGGACATCGTTTTCTCT 3’ |
| *Rara-DN* | Forward: 5’ CCCTGGAGATGGACGATGCTGAGA 3’  Reverse: 5’ TCCCCAGCATGCCTGCTATTGTCT 3’ |
| *Actb* | Forward: 5’ GTTTGAGACCTTCAACACCCC 3’  Reverse: 5’ GTGGCCATCTCCTGCTCGAAGTC 3’ |
