# Supplementary material for: Signalling Through Retinoic Acid Receptors is Required for Reprogramming of Both Mouse Embryonic Fibroblast Cells and Epiblast Stem Cells to Induced Pluripotent Stem Cells
Source: Stem Cells. 2015 Apr 23;33(5):1390–404. doi: 10.1002/stem.1926 (PMC4863141; doi:10.1002/stem.1926)
Supplement: Supplementary file 9 — Supplementary Table S2 [file STEM-33-1390-s009.docx]

| Gene Name  **Table S2 Taqman Probes for qRT-PCR** | Probe |
| --- | --- |
| *Adh1* | Mm00507711_m1 |
| *c-Myc* | Mm01192721_m1 |
| *Cyp26a1* | Mm00514486_m1 |
| *Dppa3* | Mm00836373_g1 |
| *Esrrb* | Mm00442411_m1 |
| *Fgf5* | Mm00438615_m1 |
| *Gapdh* | 4352339E |
| *Gata6* | Mm00802636_m1 |
| *Klf4* | Mm00516104_m1 |
| *Lef1* | Mm00550265_m1 |
| *Lrh1* | Mm00446088_m1 |
| *Nanog* | Mm02384862_g1 |
| *Nr0b1* | Mm00431729_m1 |
| *Oct4* | Mm00658129_gH |
| *Raldh2* | Mm00501306_m1 |
| *Rara* | Mm00436264_m1 |
| *Rarb* | Mm01319677_m1 |
| *Rarg* | Mm00441091_m1 |
| *Rex1* | Mm03053975_g |
| *Sox1* | Mm00486299_s1 |
| *Sox2* | Mm03053810_s1 |
| *Stra6* | Mm00486457_m1 |
| *T* | Mm01318252_m1 |
| *Tcf1* | Mm00493445_m1 |
