# Supplementary material for: Signalling Through Retinoic Acid Receptors is Required for Reprogramming of Both Mouse Embryonic Fibroblast Cells and Epiblast Stem Cells to Induced Pluripotent Stem Cells
Source: Stem Cells. 2015 Apr 23;33(5):1390–404. doi: 10.1002/stem.1926 (PMC4863141; doi:10.1002/stem.1926)
Supplement: Supplementary file 10 — Supplementary Table S3 [file STEM-33-1390-s010.docx]

**Table S3 Rex1GFP^+^ Colonies Reprogrammed in N2B27/LIF**

|  | **4F** | **6F** |
| --- | --- | --- |
| **Medium** | **Expt. 1 2 3 Total** | **Expt. 1 2 3 Total** |
| **D4**  **N2B27/LIF/Dox 2i/LIF** | 0; 0; 0 0 | 0; 0; 0 0 |
| **D8**  **N2B27/LIF/Dox 2i/LIF** | 0; 0; 0 0 | 5; 2; 1 8 |
| **D12**  **N2B27/LIF/Dox 2i/LIF** | 1; 0; 0 1 | 4; 5; 8 17 |
|  |  |  |

MEF reprogramming experiments in N2B27/LIF in Dox-inducible 4F or 6F.After nucleofection, MEFs were plated at 2x10^5^/well, into 3 wells of a 6-well plate for each group. Dox was withdrawn at indicated days and medium was switched from N2B27/LIF to 2i/LIF simultaneously. The colonies were scored at day 17. Expt. Experiment.
